# A young child formula supplemented with a synbiotic mixture of scGOS/lcFOS and *Bifidobacterium breve* M-16V improves the gut microbiota and iron status in healthy toddlers

**DOI:** 10.3389/fped.2024.1193027

**Published:** 2024-10-14

**Authors:** Charmaine Chew, Misa Matsuyama, Peter S. W. Davies, Rebecca J. Hill, Mark Morrison, Rocio Martin, Francisco M. Codoñer, Jan Knol, Guus Roeselers

**Affiliations:** ^1^ Danone Research & Innovation, Singapore, Singapore; ^2^ Laboratory of Microbiology, Wageningen University, Wageningen, Netherlands; ^3^ Faculty of Medicine, Child Health Research Centre, The University of Queensland, Brisbane, QLD, Australia; ^4^ Faculty of Medicine, Frazer Institute, The University of Queensland, Brisbane, QLD, Australia; ^5^ Danone Research & Innovation, Utrecht, Netherlands

**Keywords:** early life, nutrition, microbiome, probiotics, iron supplementation, milk, toddler

## Abstract

Early-life gut microbiota development depends on a highly synchronized microbial colonization process in which diet is a key regulator. Microbiota transition toward a more adult-like state in toddlerhood goes hand in hand with the transition from a milk-based diet to a family diet. Microbiota development during the first year of life has been extensively researched; however, studies during toddlerhood remain sparse. Young children's requirement for micronutrients, such as dietary iron, is higher than adults. However, their intake is usually sub-optimal based on regular dietary consumption. The Child Health and Residence Microbes (CHaRM) study, conducted as an adjunct to the GUMLi (Growing Up Milk “Lite”) trial, was a double-blind randomized controlled trial to investigate the effects on body composition of toddler milk compared to unfortified standard cow's milk in healthy children between 1 and 2 years of age in Brisbane (Australia). In this trial, fortified milk with reduced protein content and added synbiotics [*Bifidobacterium breve* M-16V, short-chain galactooligosaccharides, and long-chain fructooligosaccharides (ratio 9:1)] and micronutrients were compared to standard unfortified cow's milk. In the present study, the effects of the intervention on the gut microbiota and its relationship with iron status in toddlers were investigated in a subset of 29 children (18 in the Active group and 11 in the Control group) who completed the CHaRM study. The toddler microbiota consisted mainly of members of the phyla Firmicutes, Bacteroidota, and Actinobacteriota. The abundance of the *B. breve* species was quantified and was found to be lower in the Control group than in the Active group. Analysis of blood iron markers showed an improved iron status in the Active group. We observed a positive correlation between *Bifidobacterium* abundance and blood iron status. PICRUSt, a predictive functionality algorithm based on 16S ribosomal gene sequencing, was used to correlate potential microbial functions with iron status measurements. This analysis showed that the abundance of predicted genes encoding for enterobactin, a class of siderophores specific to *Enterobacteriaceae*, is inversely correlated with the relative abundance of members of the genus *Bifidobacterium*. These findings suggest that healthy children who consume a young child formula fortified with synbiotics as part of a healthy diet have improved iron availability and absorption in the gut and an increased abundance of *Bifidobacterium* in their gut microbiome.

## Introduction

As infants grow, many micronutrients are needed for their development. Macro- and micronutrient deficiencies increase a young child's risk of infection and can compromise growth and health trajectories (1). Iron (Fe) deficiency is considered the most frequent micronutrient deficiency and has been reported to predispose children to the aforementioned risks (2–4). Furthermore, studies have shown that an iron deficiency in infancy has a long-term impact on mental, motor, and cognitive functions later in life (5–7). This is especially important as dietary iron requirements are higher in children during the first 2 years of life and adequate iron status is a prerequisite for optimal child development, especially in toddlerhood (2).

When consumed according to the appropriate nutritional guidelines, cow's milk provides essential micro- and macronutrients to a toddler's diet, especially when transitioning from a predominantly milk-based diet to a family diet. However, cow's milk is naturally low in iron and high in calcium, which has an inhibitory effect on iron bioavailability (8). It has been widely documented that the consumption of fortified milk by infants and toddlers results in a better iron status than in children consuming cow's milk (9–11). Furthermore, cow's milk from 1 year of age is often a significant contributor to the total dietary protein intake. Rolland-Cachera et al. (12) reported that protein intake above metabolic requirements is correlated with increased secretion of growth mediators, insulin, and insulin-like growth factor I, which could enhance fat deposition and weight gain, along with increased body fat and risk of obesity in later life (13, 14). Furthermore, it has been documented that nutrient levels in usual family diets often do not fully support the micro-/macronutrients required for a child's development (15–18). It has previously been demonstrated that the consumption of suitable amounts of fortified milk as a supplement to the regular family diet is an effective source of complementary nutrition (11, 19).

De Filippo et al. (20) demonstrated that dietary fiber and protein intake variations can profoundly impact the gut microbiota in children from different countries. The functional diversity of the microbiota reflects diet and life style. This illustrated by the observation that children in rural Burkina Faso have a gut microbiome enriched in taxa known to have the capacity to break down polysaccharides, in contrast to Italian children of the same age.

Several studies have highlighted the interaction between nutrition and microbiota composition in undernutrition conditions. Some bacterial groups, such as Pseudomonadota (synonym Proteobacteria) or *Enterococcaceae* spp., have been associated with diarrhea and an overgrowth of opportunistic bacteria in malnutrition conditions (21, 22). However, such a distinctive characteristic is usually less apparent in healthy cohorts, where microbiota signatures are generally less conclusive as there are multiple factors such as inter-subject variability, diet, and genetics (23–27).

Lovell et al. (28) showed that nutrient supplementation of young child formula (YCF) with synbiotics as part of a complete diet for 12 months between the ages of 1 and 2 years resulted in significantly higher Fe, vitamin D, vitamin C, and zinc (Zn) levels in children compared to unfortified cow's milk. In addition, Matsuyama et al. (29) showed that this YCF with synbiotics facilitated the recruitment and expansion of the *Bifidobacterium* spp. among children who consumed this formula. In this study, we aim to explore the effect of synbiotics on the composition and functionality of the gut microbiome through taxonomic classification and predicted microbial metabolic pathway analyses.

## Materials and methods

### Subjects and study population

This study used a subset of samples from the Child Health and Residence Microbes (CHaRM) study (30, 31) conducted in Brisbane, Australia, selected based on the depth and quality of available DNA sequencing data. Briefly, the CHaRM study was conducted as an adjunct to the GUMLi (Growing Up Milk “Lite”) trial, a multi-center double-blind, randomized control trial (Brisbane, Australia, and Auckland, New Zealand) investigating the effect of a fortified young child formula (Active) compared to unfortified cow's milk (Control) on various outcomes in toddlers between the ages of 1 and 2 years (30).

The Active group received fortified milk supplemented with synbiotics: a formulation with 7.8 × 10^8^ cfu/100 ml of *B. breve* M-16V, 1.8 g of short-chain galactooligosaccharides (scGOS), and 0.2 g of long-chain fructooligosaccharides (lcFOS) per 100 ml (Supplementary Table 1). The participants consumed two 150 mL servings of formula daily. An energy-matched, non-fortified cow’s milk was used as a Control. In addition, the Active milk was fortified with 1.3 mg of Fe and 1.2 µg of vitamin D and vitamin C to increase the bioavailability of non-heme Fe (ferrous citrate). Further compositional differences between the Active and the Control milk are described in the study by Wall et al. (30) and the corresponding ethical approval can be found in the study by Matsuyama et al. (29).

Of the 48 children who completed the CHaRM study, a subset of 29 (18 subjects from the Active group and 11 from the Control group, details in Supplementary Table 2), for which a minimum of 15,000 high-quality sequencing reads were generated, was included in the current study.

### Blood parameters

Blood samples were collected at baseline and month 12 as described by Lovell et al. (28). In this study, we aimed to link the same parameters [serum iron, ferritin, and hemoglobin (Hb)] with the functional analyses of the gut microbiome.

### Fecal DNA isolation and sequencing

All fecal samples were collected and preserved, and gut microbiota DNA was extracted as described previously (31). Briefly, a repeated beat-beating and a column (RBB + C) technique was used based on Yu and Morrison (32) and modified to suit the automated Maxwell 16MDx system (Promega, Madison, WI, USA). An aliquot of each fecal sample was mixed with beads, homogenized with lysis buffer, and homogenized in a Precellys 24 homogenizer (Bertin Corp, Rockville, MD, USA). After the protocol was completed, the supernatant was transferred into Maxwell 16 MDx cartridges for elution. At this step, a non-template control was used to check the quality of each batch of buffers (lysis and elution). DNA samples were normalized to sample concentration before the creation of 16S rRNA gene amplicon libraries following Illumina library preparation instructions, using the primers of Klindworth et al. (33) to amplify the V3-V4 hypervariable regions of the bacterial 16s rRNA gene region. The libraries were then sequenced for 300 paired-end cycles using the Illumina MiSeq platform.

### Quantitative polymerase chain reaction

In parallel, we used four targeted quantitative polymerase chain reaction (q-PCR) assays to detect total bacteria, total *Bifidobacterium*, *B. breve* group, and *B. breve* M-16V strain. Briefly, the normalized DNA samples were used for all the amplified quantitative real-time PCR amplification performed using an ABI Prism 7900HT (Applied Biosystems, California, USA) using the TaqMan Universal Master Mix (Applied Biosystems, Austin, TX, USA). Standards for each target were generated from their genomic DNA and were amplified with specific primers, and purified using a MinElute PCR Purification Kit (Qiagen, Valencia, CA, USA) as per the manufacturer’s protocol. Final standard concentrations were quantified using a NanoDrop 2000 (Thermo Scientific, Waltham, MA, USA). For a detailed description of the methods, including the oligonucleotide sequences of the primers and probes used for the q-PCR analyses, please see Chua et al. (34).

Samples were prepared using a Microlab NIMBUS (Hamilton Robotics, Reno, Nevada, USA) in a customized setup. The q-PCR conditions were 1 cycle of 95°C for 20 s, followed by amplification at 95°C for 1 s, and 62°C for 20s for 40 cycles, and then 1 cycle of 95°C for 15 s, followed by 60°C for 15 s, and 95°C for 15 s. SDS 2.4 (Applied Biosystems) was used to visualize and check the abnormality of the curves deviating from standard amplification. Raw data were then exported into Microsoft Excel, where the Ct values were transformed to log copy numbers per gram of feces for the statistical analyses.

### 16S rRNA gene sequencing analysis

The data analysis performed in this sub-study differs from that of Matsuyama et al. (29) by using a higher sequence coverage cut-off and optimized sequence data quality control. Briefly, pre-processing and filtering of the raw 16S rRNA gene sequence data resulted in an average of 18,916 high-quality reads per sample, and each rarefaction analysis showed slopes reaching a plateau, indicating that a sufficient sequencing depth had been achieved. An adaptation of the “Quantitative Insights into Microbial Ecology” (QIIME) v1.9.1 package (35) was used for the processing and analysis as described in (29). The paired-end reads were demultiplexed and trimmed (q > 20) before being merged using QIIME. These were dereplicated and counted using mothur (36) and reads with a low abundance (less than 2 reads across all samples) were discarded. Chimeras were removed using VSEARCH (37), using the RDP gold database (38) as a reference. Reads that contained PhiX or adapters as defined in Deblur (part of QIIME2) (39) were eliminated. Taxonomic assignment was performed using the RDP classifier (40) against the SILVA_138.1 database (41), from which results where the sequences were aggregated at the genus and phylum level, were further explored. Reads with eukaryotic assignments and reads with a low relative abundance of up to 0.0005% across all samples were excluded from further downstream analysis. Samples were rarefied, and α-diversity was calculated using the phyloseq (42) and vegan (43) packages in R software (version 3.5.1) (44).

In addition to the amplicon-based analysis, we used a metagenome functional content bioinformatics package that provides potential functional category abundances for a microbial community from marker genes extracted from full genomes using 16S operational taxonomic unit (OTU) profiles as implemented in the PICRUSt software package (45) using the default parameter. Briefly, the 16S OTUs were normalized by copy number and mapped onto a phylogenetic tree created from an ancestral state reconstruction (ASR) reference genome database with more than 10× coverage. The predicted metagenome functional abundance counts per sample were then collapsed into functional categories based on KEGG Gene Orthology pathways for analyses. We investigated the change in predicted functionality abundances using the delta difference in the counts at baseline and at month 12.

### Statistical analysis

PRISM GraphPad 8 (version 8.4.3) was used for all the statistical analyses. The change from baseline within each group was analyzed using a non-parametric pair-wise Mann–Whitney test. The baseline and end-of-intervention parameters of the groups were compared using a non-parametric Wilcoxon test. The effect size was calculated using Cohen's d, where the mean difference between the two groups was divided by the pooled standard deviation. The interaction of the intestinal microbiota composition with the intervention was visualized spatially using distance matrices in the principal component analysis (PCA), an unconstrained ordination method, using CANOCO 5 (2012) software. The Benjamini–Hochberg procedure (46) was used to reduce the false discovery rate (FDR) for all comparisons (within each group, at baseline, and at the end of the intervention) made using the predicted microbial functionalities.

## Results

In this subset study, the participants were evenly divided by gender and anthropometric measurements, such as weight and length at baseline, with no significant differences observed (Supplementary Table 1). At the end of the intervention, there were significant differences as noted in previous publications (28–30).

### Blood parameters

**Figure 1:**
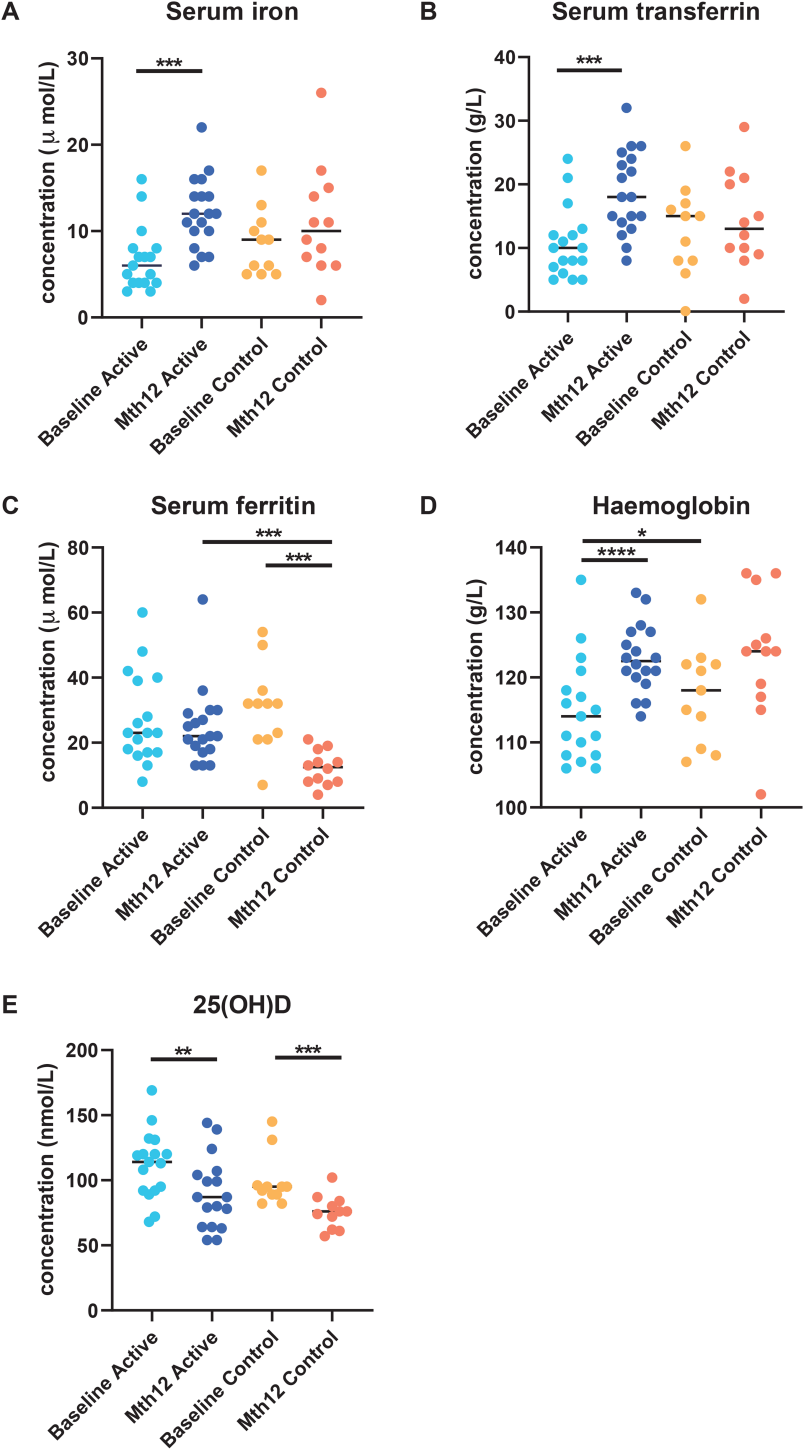
Blood serum parameters **(A)** serum iron, **(B)** serum ferritin, **(C)** serum transferrin, **(D)** hemoglobin, and **(E)** 25(OH)D were measured at baseline and at the end of the intervention (month 12) in participants in both groups as reported previously by Lovell et al. (28). Serum iron **(A)** and transferrin **(C)** increased in the Active group, while no significant difference was observed in the Control group. A significant decrease in serum ferritin **(B)** was observed between baseline and month 12 in the Control group. At month 12, serum ferritin was significantly lower in the Control group than in the Active group.

The same findings in the main GUMLi study were observed in this subset of samples from the CHaRM study. There was no statistically significant difference in serum iron at baseline between the groups (*p* = 0.111), and significant differences were detected at the end of the study within the Active group (*p* < 0.0008, effect size d = 1.390574) but not within the Control group (*p* = 0.898) (Figure 1). Similarly, there was no difference at baseline between the groups for transferrin, 25(OH)D and ferritin, (*p* > 0.05) amongst this subset of samples (data not shown). We did not observe a significant difference between the groups in hemoglobin levels at baseline (*p* = 0.346) or at month 12 (*p* = 0.580).

The hemoglobin levels were significantly increased from baseline to month 12 in the Active group (*p* < 0.0001, effect size = 1.125876), while it was significant in the Control group for the same comparison (*p* = 0.024). The Vitamin D indicator, 25(OH)D, was significantly reduced from baseline to month 12 in both the Active (*p* = 0.003, effect size = 0.826) and the Control groups (*p* = 0.001, effect size d = 1.399) although they were still within the healthy range according to the Endocrine Society’s Clinical Practice Guidelines (>50 nmol/L) (47).

At the end of the study, there were significantly higher transferrin levels in the Active group from baseline to month 12 (*p* = 0.0029) compared to the Control group from baseline to month 12 (*p* = 0.527). In addition, serum ferritin concentration was significantly lower at month 12 compared to the baseline in the Control group (*p* = 0.001). A significant difference was only observed at month 12 between the Active and Control groups (*p* = 0.0002) (Figure 1).

### q-PCR

We quantified the total bacteria [copy number in feces (log_10_/g)] in the fecal samples and observed an increase that was significantly different from baseline to month 12 in the Active group [mean difference 0.84 (log_10_/g), *p* = 0.001] and in the Control group [mean difference 0.30 (log_10_/g), *p* = 0.032]. No significant difference was observed at baseline or at month 12 between the intervention groups. We observed no significant difference in the total count of *Bifidobacterium* copy number in feces (log_10_/g) at baseline or at month 12 between the Active and Control groups. There was a significant decrease in the *B. breve* group in the Control group from baseline to month 12 [0.79 (log_10_/g), *p* = 0.049]. This decrease was not observed in the Active group. The supplemented probiotic strain, *B. breve* M-16V, was only detected in the Active group and we observed a significant increase [1.66 (log_10_/g), *p* ≤ 0.001] between baseline and month 12 (Supplementary Figure 1).

### 16S rRNA gene profiling and functionality predictions

The major bacterial phyla present in this subset of samples were the same as previously reported (29), namely, *Firmicutes*, *Bacteroidetes*, and *Actinobacteria*, characteristic of healthy human gut microbiota. Increased α-diversity among this subset of samples was also demonstrated (Supplementary Figure 2). Here, we report the most abundant bacteria genera showing significant differences between baseline and month 12. *Bifidobacterium* decreased in the Control group from 20.6% at baseline to 7.9% at month 12 (*p* = 0.020). The *Escherichia-Shigella* group significantly decreased during the intervention in the Active group (from 4.17% to 0.18%) (*p* = 0.0017) and the Control group (from 1.8% to 0.30%) (*p* = 0.004) (Supplementary Figures 3A,C). For the other genera investigated, such as *Bacteroides*, *Collinsella*, and *Faecalibacterium*, no significant differences were observed (Supplementary Figure 3). Furthermore, the genus *Veillonella* showed a significant decrease as observed previously (29), most noticeably in the Active group.

**Figure 2:**
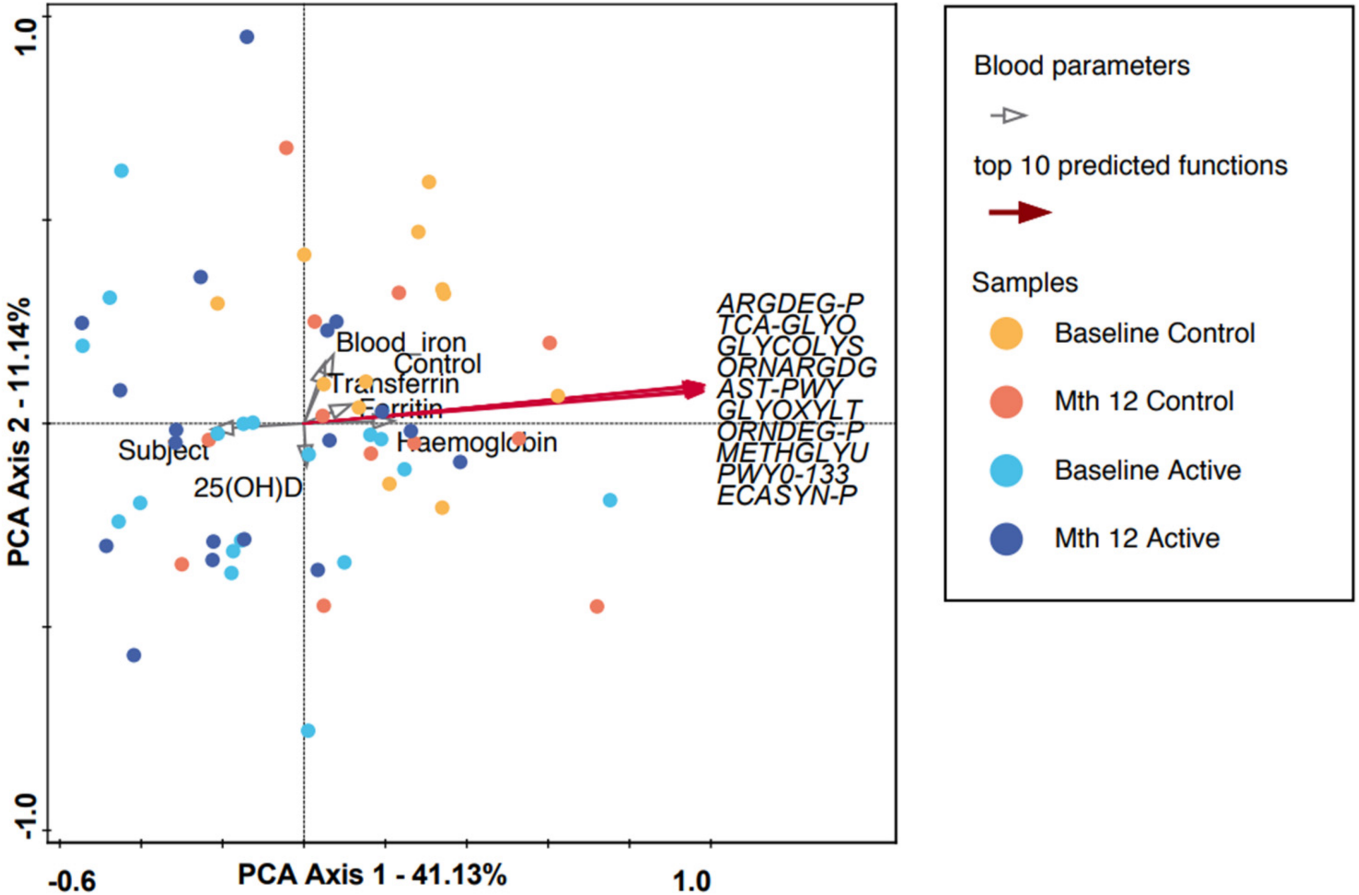
A PCA plot in which each dot represents a microbiota composition sample. Blue and purple indicate the Active group at baseline and month 12, and red and yellow indicate the Control group at baseline and month 12, respectively. PCA axis 1 shows an explained variance of 41.13% with a strong correlation with the top 10 predicted microbial functions (using the PICRUSt algorithm, refer to Supplementary Table 3), while the measured blood parameters are represented by gray clear arrows. ARGDEG-PWY, super-pathway of L-arginine, putrescine, and 4-aminobutanoate degradation; AST-PWY, L-arginine degradation II (AST pathway); ECASYN-PWY, enterobacterial common antigen biosynthesis; ENTBACSYN-PWY, enterobactin biosynthesis; GLYCOL-GLYOXDEG-PWY, super-pathway of glycol metabolism and degradation; GLYOXYLATE-BYPASS, glyoxylate cycle; METHGLYUT-PWY, super-pathway of methylglyoxal degradation; ORNARGDEG-PWY, super-pathway of L-arginine and L-ornithine degradation; ORNDEG-PWY, super-pathway of ornithine degradation; PWY0-1338, polymyxin resistance; TCA-GLYOX-BYPASS, super-pathway of glyoxylate bypass and TCA.

In Figure *2*, the 10 most abundant predicted functionalities were subjected to PCA. PCA Axis 1 explained 41.13% of the variation, with the most significant predicted functions pointing (red arrows) to the right quadrants. However, the majority of the samples, regardless of group or timepoint, congregated and overlapped in the center of the graph. Functional abundance predicted by PICRUSt was compared between the intervention groups at either baseline, month 12 or within each intervention group from baseline to month 12. No significant differences were observed at baseline or month 12 after applying multiple correction (FDR). In the Active (baseline to month 12) and Control group comparison, several functions remained significant after FDR correction (refer to Supplementary Table 3). Among the listed functions, enterobactin biosynthesis was reduced in both the Active (*p* = 0.00182) and Control (*p* = 0.047) groups at the end of the study.

**Figure 3:**
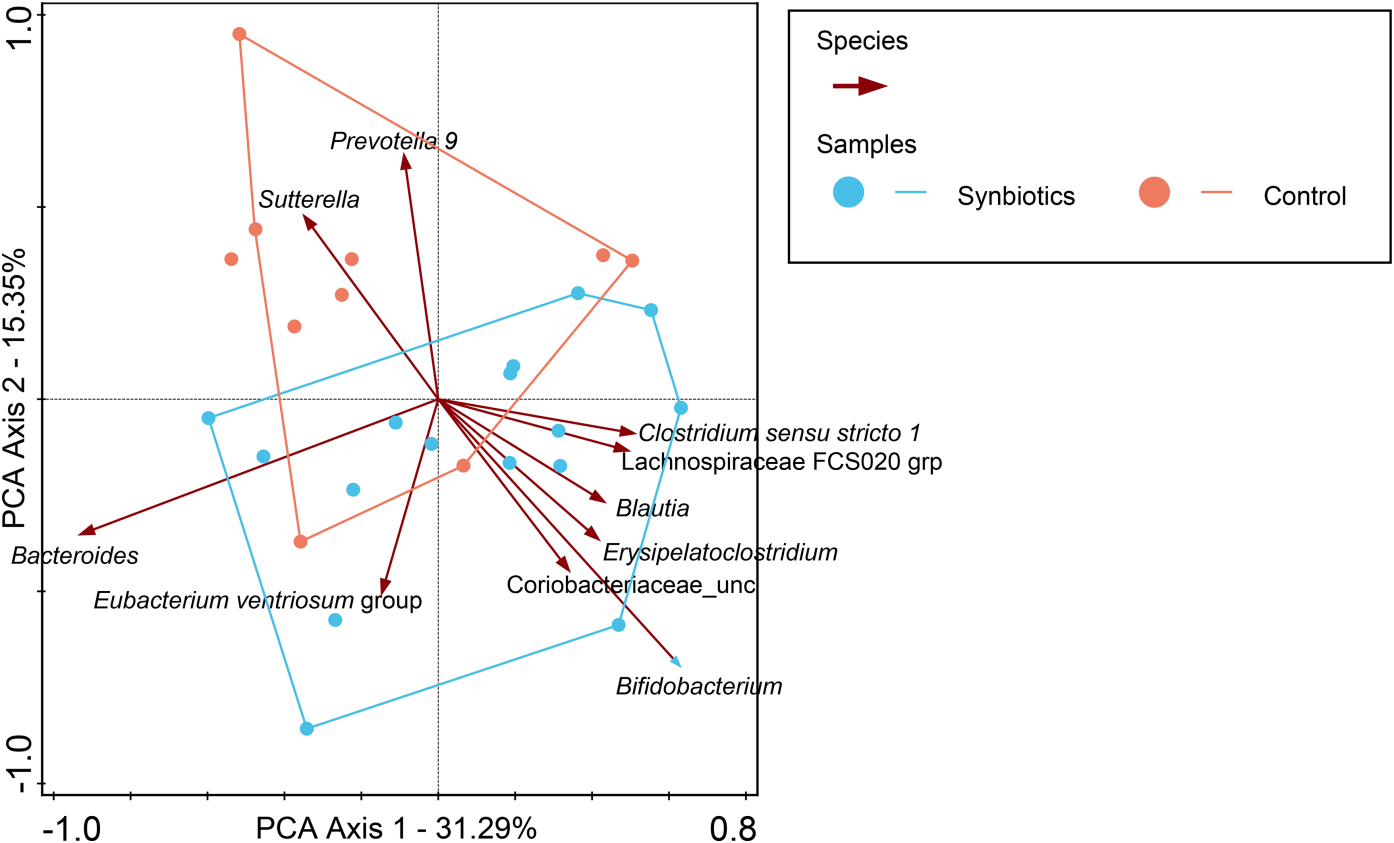
In this PCA coordinate plot, the changes in relative abundance from baseline for each sample (month 12 to baseline) were used. From the plot, it can be observed that *Bifidobacterium* is one of the most influential factors driving the synbiotics group cluster, while in the Control group, it is *Prevotella* 9 and *Sutterella*.

**Figure 4:**
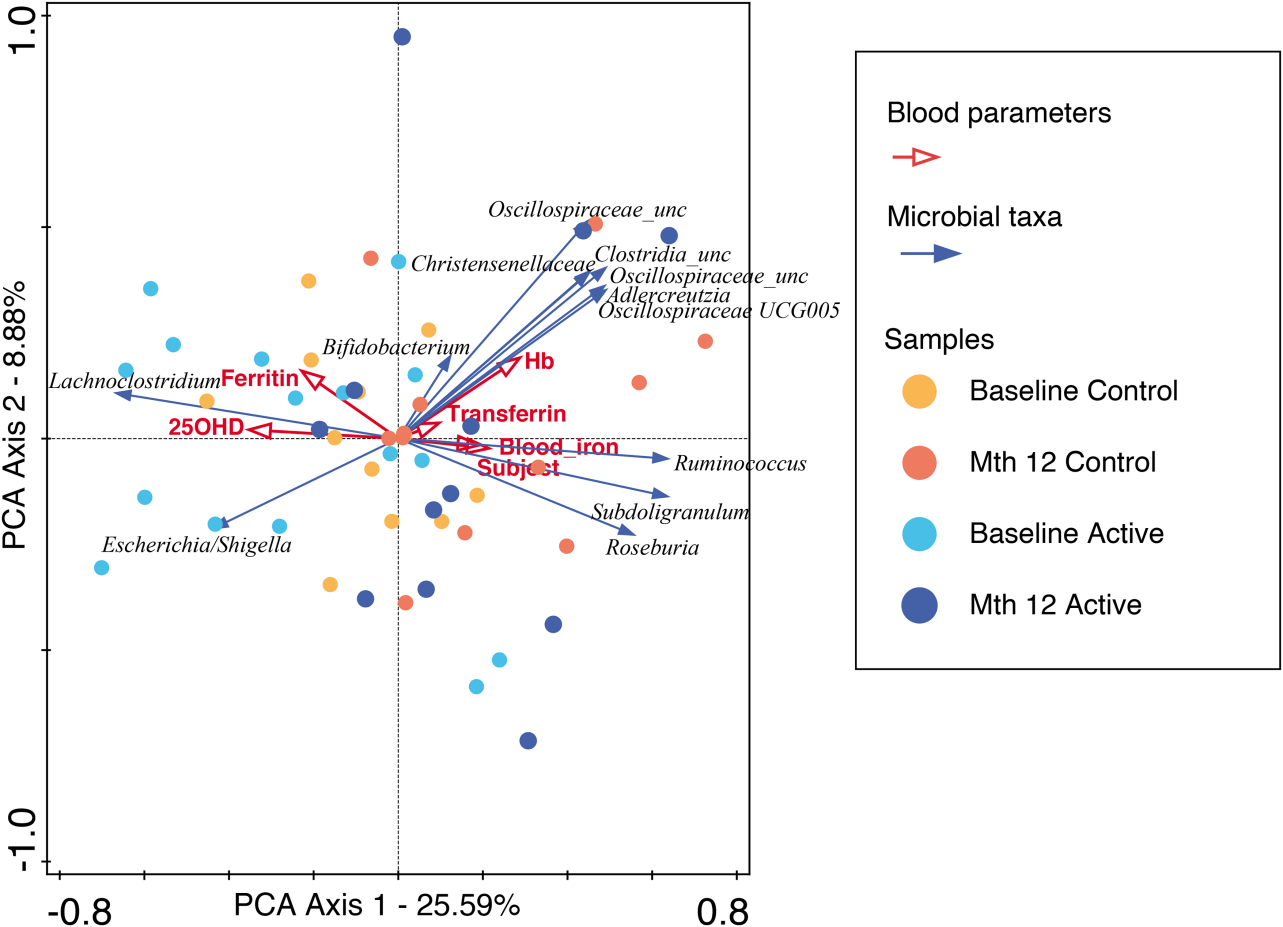
An unconstrained PCA plot of all the samples using the predicted functionalities as the supplementary variables. The blood parameters are denoted by red arrows and the top 10 bacteria genera by blue arrows. The baseline samples from both the Active and Control groups are predominantly in the left quadrants with *Escherichia-Shigella* explaining the separation, while the month 12 samples are in the center to right quadrants with blood iron, transferrin, and hemoglobin, along with other bacteria genera, showing temporal changes across the intervention.

For participants in the Active group at month 12, we observed a trend toward the right quadrant of the PCA plot that was in line with the explained genera and blood parameters (iron, transferrin, and Hb). This separation explained 25.41% of the observed variation in the microbiomes during the intervention (in Supplementary Figure 4). In the Control group, every blood parameter except hemoglobin trended in the opposite direction, although the bacteria genera were also shifted toward the participants at month 12, explaining 46.95% of the variation observed. In addition, *Bifidobacterium* pointed in the opposite direction (toward baseline), while the remaining top 10 genera pointed toward month 12, indicating a possible normal microbiota maturation with age. We evaluated the influence of the intervention on the microbiota over time by using the delta change in the relative abundance from baseline to month 12. The PCA analysis of the 10 most abundant bacteria genera explained 31.29% of the observed variation, as shown in Figure 3. The figure shows that the clustering in the Active group was mainly driven by *Bifidobacterium* and *Bacteroides*. The clustering in the Control group was mainly driven by *Prevotella* 9 and *Sutterella*. A combined unconstrained PCA plot is presented in Figure 4. PCA axis 1 explained 25.59% of the variation seen in the microbiota, with the baseline samples clustered in the bottom left quadrant of the plot. The *Escherichia-Shigella* group pointed in the opposite direction to the other influencing genera that clustered at month 12 (for both Active and Control group samples). On the same plot, serum ferritin and 250HD pointed toward the left quadrants (higher levels at baseline) while serum iron, transferrin, and hemoglobin pointed toward the month 12 samples (increased levels at the end of the intervention). This is consistent with the results shown in Figure 1.

## Discussion

In this study, we further explored the potential effects of a year-long intervention with YCF supplemented with synbiotics on the gut microbiota composition and predicted the microbial functionality in a subset of children who participated in the CHaRM study (29).

At the end of the intervention, we observed significant differences in several blood parameters; serum iron, transferrin, and hemoglobin levels were higher in the Active group at month 12 compared to baseline. The Control group showed smaller, significant changes for the same corresponding parameters. Serum iron levels were higher in the Active group at month 12. This suggests that increased iron uptake coincided with increased iron carriers (transferrin) in the blood and ferritin stores were maintained in the body.

Iron is an important micronutrient that plays a role in the normal development of mental and motor skills in children (5, 7). In developing countries, the prevalence of iron deficiency in early life is high. However, two recent iron fortification trials in infants in developing countries have raised safety concerns: in Ghana, there was an increased rate of hospitalization possibly due to diarrhea (48), and in Pakistan, a small but significant increase was reported in the overall prevalence of diarrhea (49).

At the gut microbiota level, iron supplementation has been associated with adverse effects such as an increase in opportunistic pathogenic bacteria and a decrease in beneficial bacterial taxa such as *Bifidobacteriaceae* and *Lactobacillaceae* when supplemented individually (50–53). Nevertheless, it is still imperative to provide adequate iron-rich complementary foods in the diets of infants and children (54). This supports the rationale for including iron supplementation in YCF to provide sufficient sources of iron for young children. Unfortunately, iron absorption from iron-fortified foods or oral iron supplements is often <20% (55); thus, the majority of the iron passes unabsorbed into the colon. This can adversely favor the growth of potential enteropathogens because iron is a growth-limiting nutrient for the majority of enteric Gram-negative bacteria (e.g., pathogenic *Escherichia coli* and *Salmonella*), as iron acquisition is essential for virulence and colonization.

Several studies have shown modulation of the colonic microbiota by pre-, pro-, or synbiotics can mitigate these adverse effects of iron fortification on the gut microbiome (56, 57). This study confirms changes in both the taxonomic composition and the relative abundances of predicted microbial functions over 1 year, indicative of a developing early-life microbiome. The synbiotic intervention resulted in the maintenance of *B. breve* abundances and did not appear to diminish or stagnate the growth of other bacterial groups. We observed a decrease in *B. breve* in the Control group while q-PCR quantification showed no difference in *Bifidobacterium.* However, 16S rRNA sequence data showed a decrease in the total relative abundance of the genus *Bifidobacterium*. This discrepancy between the results for *Bifidobacterium* generated by q-PCR and 16S rRNA gene analysis may be attributed in part to the different amplification and normalization biases, as previously reported by Zemb et al. (58). The presence of the supplemented probiotic strain (*B. breve* M-16 V) was only detected in the Active group, as shown by the q-PCR results. *Bifidobacterium* is one of the most abundant genera in the gut of breastfed infants and is considered a true “keystone” taxon with a strong eco-physiological impact on the microbiota composition. Therefore, the abundance of *Bifidobacterium* spp. may serve as a marker of healthy microbiota development and breastfeeding practices (59).

In this study, we observed no other differences in taxonomic composition between the Active and Control groups. Previously, infant formula with *B. breve* M-16V + scGOS/lcFOS (9:1) was reported to result in a lower fecal pH through the production of acetate. *B. breve* UCC2003 was reported to secrete iron-binding domains and ferric uptake regulatory proteins (60). It has been proposed that this is an adaptive mechanism to secure the iron required for growth and inhibit potential pathogens that are susceptible to a lower pH (61, 62), and to promote other beneficial bacteria such as butyrate producers through cross-feeding *in vitro* cultures (63, 64). We observed that *Escherichia-Shigella* was inversely correlated with butyrate-producing bacteria (e.g., members of the *Eubacterium* groups, and the genera *Ruminococcus* and *Subdoligranulum*) and blood biomarkers (serum iron, transferrin, and hemoglobin) (see Figure 3).

We are aware that these findings are only reflective of a small cohort and emphasize caution in the interpretation of the data as the children in this study were clinically healthy. We hypothesize that the mode of action by which *Bifidobacterium* may improve host iron bioavailability is by (1) lowering the colonic luminal pH and (2) converting Fe^3+^ to Fe^2+^, due to their ferric-reducing activity. In addition, *Bifidobacteria* may prevent opportunistic pathogenic bacteria from utilizing the scarce amount of iron either by (1) competitive exclusion or by (2) reducing Fe^2+^ accessibility for other microorganisms by competitively binding it to their extracellular membranes (60, 65–67). Several studies have demonstrated that iron supplementation resulted in a less detrimental effect on the microbiota when complemented with prebiotics, probiotics, or synbiotics (56, 57, 68–71).

The 10 most abundant predicted microbial functions included enterobactin biosynthesis, enterobacterial common antigen biosynthesis, and polymyxin resistance genes (see Supplementary Table 3). These pathways are adaptations that allow opportunistic pathogenic bacteria such as *Enterobacteriaceae* to grow under iron-limited conditions but the metabolic cost of carrying the genes to synthesize microbial iron chelators such as siderophores is significant. The presence of these functions suggests that iron is a limitation for potential opportunistic pathogens to thrive. In this study, healthy children who underwent the year-long intervention with a synbiotic formula or a cow's milk control formula showed a reduction in *Escherichia-Shigella* and enterobactin biosynthesis function, which provided insight into gut microbiota maturation over time. In the Active group, in addition to the microbial maturation, improved blood iron status markers were observed. This appears to support the current hypothesis that microbiome modulation in combination with iron supplementation can improve absorption and utilization and reduce the risk of iron deficiency anemia, as proposed in other studies (72–74).

Toddlerhood is an important phase in life in which adequate nutrition with micro- and macronutrients is essential for growth and development. This study has provided us with a working hypothesis on the effect of synbiotics on toddler gut microbiota composition and microbial function. In the presence of synbiotics, iron absorption may be improved through the acidification of the gut. As the study aimed to compare the effects of synbiotic-supplemented formula with cow's milk, which is a common practice, we were unable to independently differentiate the effects of iron fortification from the interaction of synbiotics with the microbiota composition. We propose that this hypothesis should be addressed using existing publicly available studies and in future clinical trials.

## Data Availability

The original contributions presented in the study are publicly available. These data can be found here: European Nucleotide Archive (ENA) under accession number PRJEB66204 (https://www.ebi.ac.uk/ena/browser/view/PRJEB66204).

## Supplementary material

Supplementary Table 1 Nutritional composition of the formula used in the trial.

Supplementary Table 2 Anthropometric measurements of the participants included in this study.

Supplementary Table 3 Comparisons generated within groups/timepoints using the PICRUSt generated functionalities with FDR corrections.

Supplementary Figure 1 q-PCR quantified log scale copy number of the various targets with median center bars.

Supplementary Figure 2 α-diversity showing (**A**) Shannon and (**B**) Simpson indices showing an increase in diversity over time.

Supplementary Figure 3 Relative abundance of several genera was found to have changed by the end of the intervention.

Supplementary Figure 4 PCA analyses showing the top 10 most abundant genera contributing to the microbiota of participants in the Active (right) and Control (left) groups. *Bifidobacterium* was enforced in the graphs. The Control group had more opportunistic pathogens associated with it at the end of the intervention compared to the Active (synbiotics) group.
