# Supplementary material for: A young child formula supplemented with a synbiotic mixture of scGOS/lcFOS and Bifidobacterium breve M-16V improves the gut microbiota and iron status in healthy toddlers
Source: Front Pediatr. 2024 Oct 14;12:1193027. doi: 10.3389/fped.2024.1193027 (PMC11513326; doi:10.3389/fped.2024.1193027)
Supplement: Supplementary Table 2 — Anthropometric measurements of the participants included in this study. [file Datasheet1.docx]

Supplementary material

**Supplementary Table 1** Nutritional composition of formula used in the trial

| Composition^1^ | Control (CM) | Active (GUMLi) |
| --- | --- | --- |
| Energy, kJ | 245 | 249 |
| Macronutrients, g |  |  |
| Protein | 3.1 | 1.7 |
| Carbohydrate | 4.5 | 7.8 |
| Total fat | 3.1 | 1.9 |
| Saturated fat | 1.9 | 1.3 |
| Total n–3 long-chain FAs (DHA+EPA+DPA), g | <0.002 | 0.04 |
| Dietary fibre, g |  |  |
| scGOS | 0 | 1.8 |
| lcFOS | 0 | 0.2 |
| Micronutrients |  |  |
| Nonheme iron, mg | 0 | 1.3 |
| Cholecalciferol, μg | 0.1 | 1.2 |

^1^Values obtained from the manufacturer (Danone) and are based on average
totals from 3 batches produced for use in the GUMLi Trial. CM, cow milk; DPA,
docosapentaenoic acid; GUMLi, Growing-Up Milk–Lite; lcFOS, long-chain fructooligosaccharides;
scGOS, short-chain galacto-oligosaccharides

**Supplementary Table 2** Anthropometric measurements of subjects included in this study

|  |  | Control | | Active | |
| --- | --- | --- | --- | --- | --- |
|  |  |  |  |  |  |
| No of subjects (n) |  | 11 | | 18 | |
| Gender F/M(total) |  | 6/5 (11) | | 9/9 (18) | |
| Average (Min-Max) |  |  |  |  |  |
| Weight | Baseline | 9.863 (8.41-11.7) | | 9.593 (7.9-11.3) | |
|  | Month 12 | 13.21 (11.1-15.3) | | 12.46 (10.6-15.2) | |
| Length | Baseline | 74.89 (71.5-79.67) | | 74.44 (71-80.5) | |
|  | Month 12 | 87.89 (85-91.5) | | 86.92 (82.5-92.5) | |
| BMI | Baseline | 17.55 (16.45-19.07) | | 17.29 (14.88-19.21) | |
|  | Month 12 | 17.06 (15.26-18.31) | | 16.48 (13.98-18.52) | |
| Fat Mass | Baseline | 2.846 (1.28-4.234) | | 3.005 (1.586-4.007) | |
|  | Month 12 | 3.98 (3.197-5.205) | | 3.476 (1.443-4.965) | |
