# Supplementary figures and images for: A young child formula supplemented with a synbiotic mixture of scGOS/lcFOS and Bifidobacterium breve M-16V improves the gut microbiota and iron status in healthy toddlers
Source: Front Pediatr. 2024 Oct 14;12:1193027. doi: 10.3389/fped.2024.1193027 (PMC11513326; doi:10.3389/fped.2024.1193027)

**A**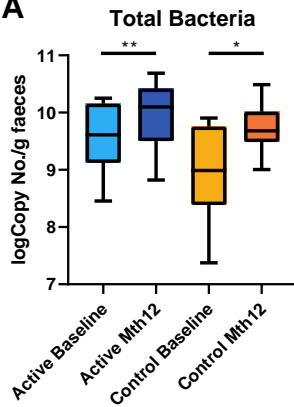**B**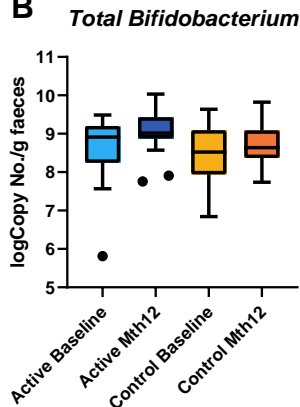**C**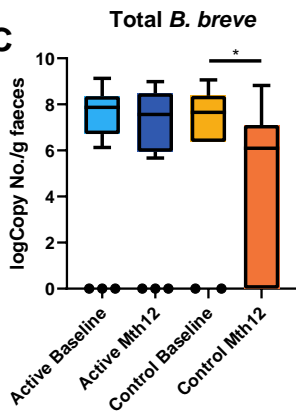**D**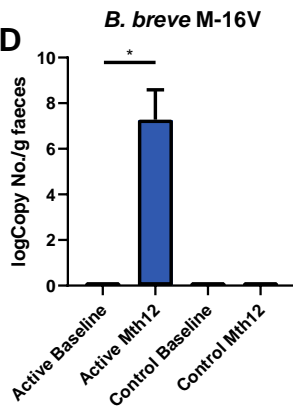

Supplement: Supplementary Figure 1 — q-PCR quantified log scale copy number of the various targets with median center bars. [file Image1.pdf]

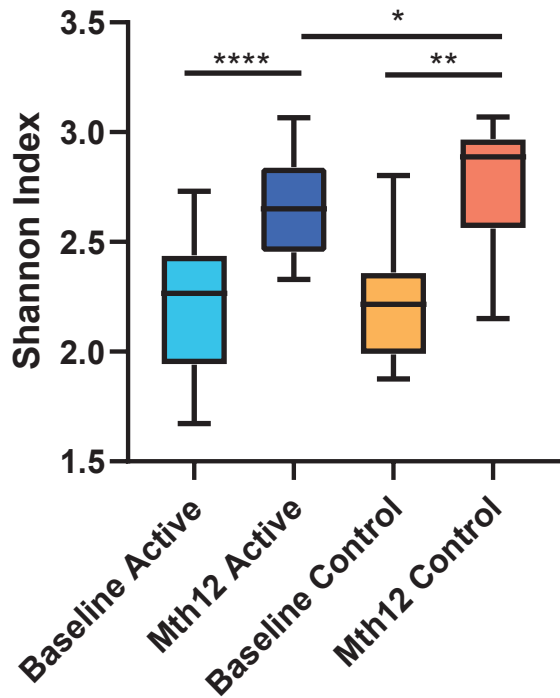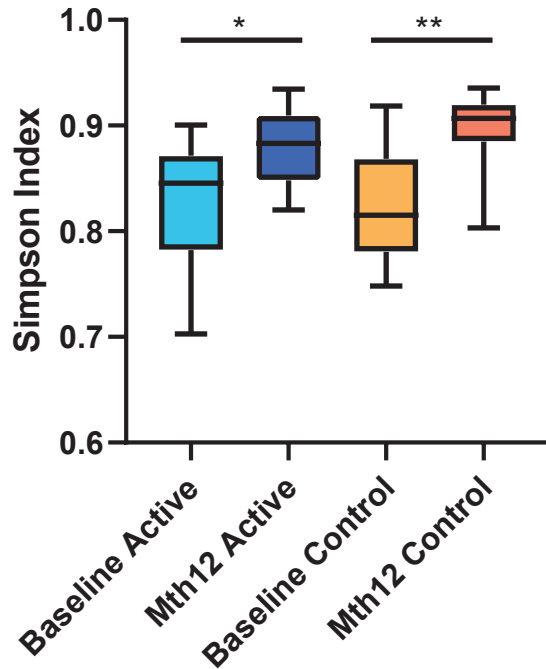

Supplement: Supplementary Figure 2 — α-diversity showing (A) Shannon and (B) Simpson indices showing an increase in diversity over time. [file Image2.pdf]

A

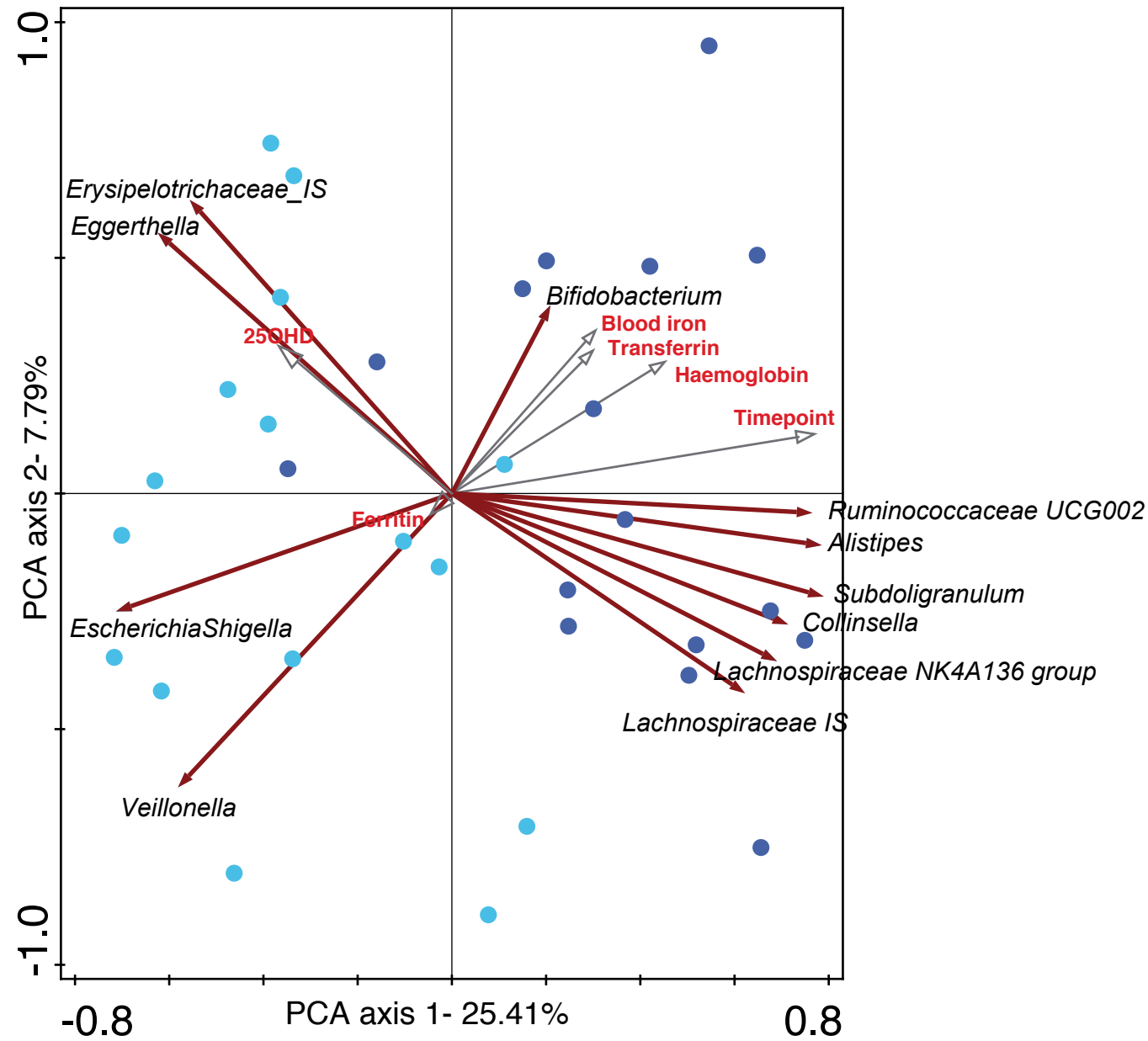

B

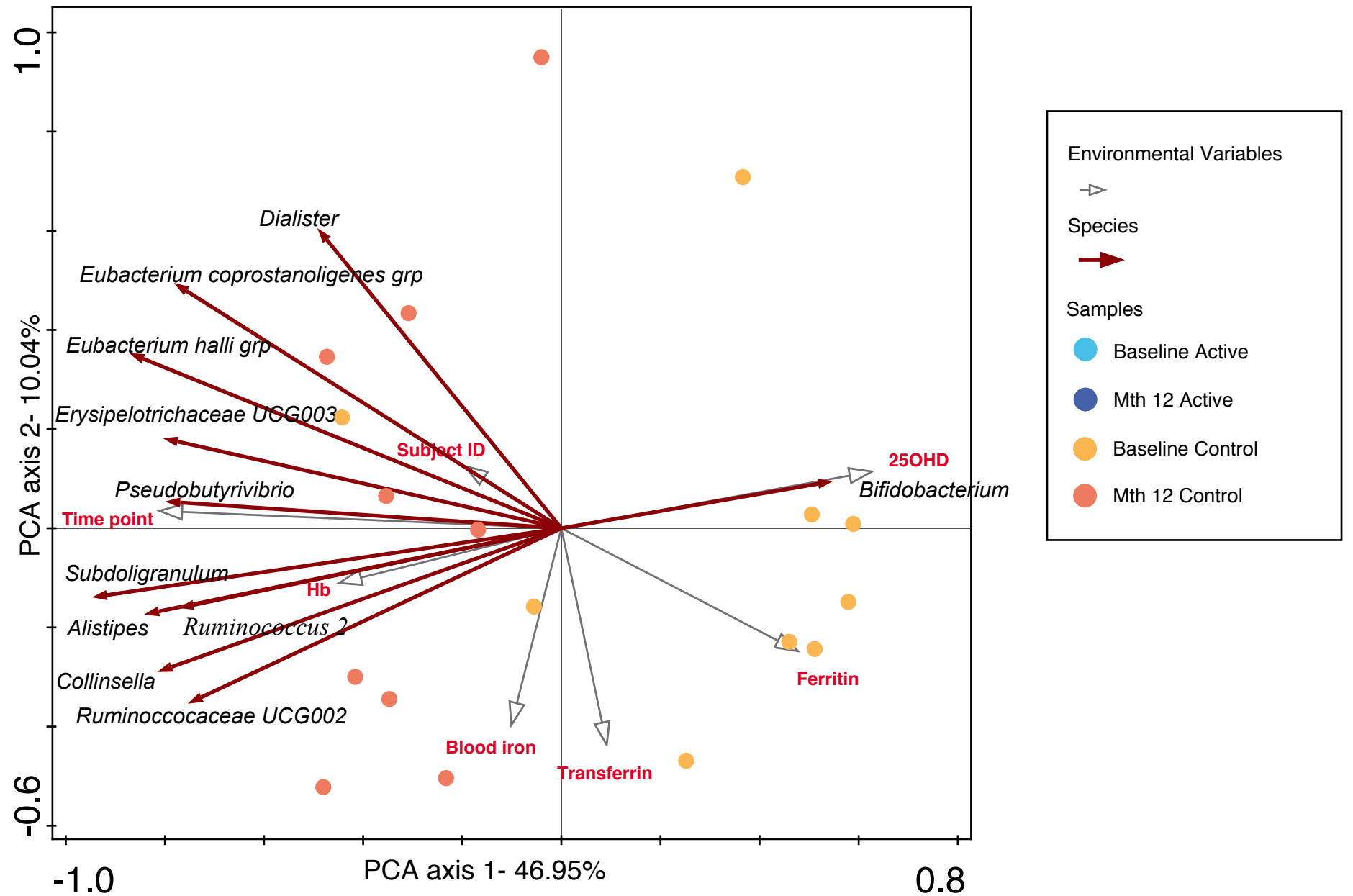

Supplement: Supplementary Figure 4 — PCA analyses showing the top 10 most abundant genera contributing to the microbiota of participants in the Active (right) and Control (left) groups. Bifidobacterium was enforced in the graphs. The Control group had more opportunistic pathogens associated with it at the end of the intervention compared to the Active (synbiotics) group. [file Image4.pdf]
