# Supplementary material for: A young child formula supplemented with a synbiotic mixture of scGOS/lcFOS and Bifidobacterium breve M-16V improves the gut microbiota and iron status in healthy toddlers
Source: Front Pediatr. 2024 Oct 14;12:1193027. doi: 10.3389/fped.2024.1193027 (PMC11513326; doi:10.3389/fped.2024.1193027)
Supplement: Supplementary Figure 3 — Relative abundance of several genera was found to have changed by the end of the intervention. [file Image3.pdf]

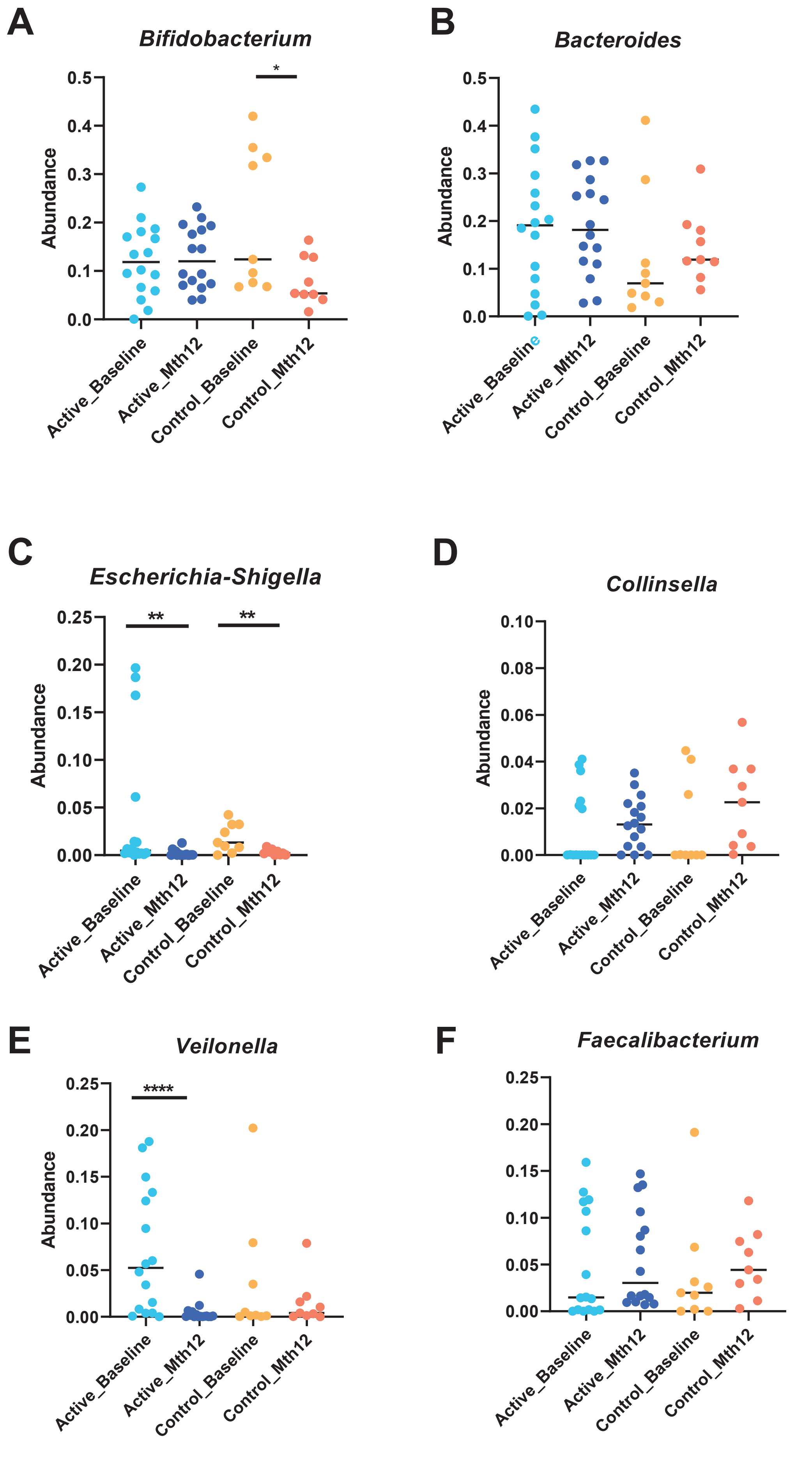
A
Bifidobacterium
Abundance
0.5
0.4
0.3
0.2
0.1
0.0
*
Active_Baseline
Active_Mth12
Control_Baseline
Control_Mth12
B
Bacteroides
Abundance
0.5
0.4
0.3
0.2
0.1
0.0
Active_Baseline
Active_Mth12
Control_Baseline
Control_Mth12
C
Escherichia-Shigella
Abundance
0.25
0.20
0.15
0.10
0.05
0.00
**
**
Active_Baseline
Active_Mth12
Control_Baseline
Control_Mth12
D
Collinsella
Abundance
0.10
0.08
0.06
0.04
0.02
0.00
Active_Baseline
Active_Mth12
Control_Baseline
Control_Mth12
E
Veilonella
Abundance
0.25
0.20
0.15
0.10
0.05
0.00
****
Active_Baseline
Active_Mth12
Control_Baseline
Control_Mth12
F
Faecalibacterium
0.25
0.20
0.15
0.10
0.05
0.00
Active_Baseline
Active_Mth12
Control_Baseline
Control_Mth12
